# The Regulatory T-cell Transcription Factor Foxp3 Protects against Crescentic Glomerulonephritis

**DOI:** 10.1038/s41598-017-01515-8

**Published:** 2017-05-03

**Authors:** Chen Yang, Xiao-Ru Huang, Erik Fung, Hua-Feng Liu, Hui-Yao Lan

**Affiliations:** 1 Institute of Nephrology, Guangdong Medical University, Zhanjiang, Guangdong China; 2 Department of Medicine and Therapeutics, and Li Ka Shing Institute of Health Sciences, and Shenzhen Research Institute, The Chinese University of Hong Kong, Hong Kong, China

## Abstract

Regulatory T cells (Tregs) have been shown to play a protective role in glomerulonephritis (GN) and Foxp3 is a master transcription factor in Treg development. In this study, we examined the functional role and mechanisms of Foxp3 in a mouse model of accelerated anti-glomerular basement membrane (anti-GBM) GN induced in antigen-primed Foxp3 transgenic (Tg) mice. Compared with littermate of wildtype (WT) mice in which induced severe crescentic GN developed with progressive renal dysfunction, Foxp3 Tg mice had reduced crescent formation, urinary protein excretion, plasma creatinine and decline in creatinine clearance. The protective role of Foxp3 in crescentic GN was associated with a markedly suppressed expression of proinflammatory interleukin-1 beta (IL-1β), tumour necrosis factor-alpha (TNF-α) and monocyte chemoattractant protein 1 (MCP-1), and diminished infiltration of the kidneys by CD3^+^ T cells and F4/80^+^ macrophages. Moreover, overexpression of Foxp3 resulted in a significant increase in CD4^+^ Foxp3^+^ Tregs systemically and in the diseased kidneys, thereby blunting Th1, Th2, and Th17 responses systemically and in the kidneys. In conclusion, Foxp3 protects against kidney injury in crescentic GN through enhancement of Treg numbers and function, and suppression of Th1, Th2 and Th17 immune responses at the systemic and local tissue levels.

## Introduction

Glomerulonephritis (GN) is a common cause of chronic kidney disease and end stage renal disease driven by a misdirected immune response^1^. Studies have shown that CD4^+^ T cells play a pivotal role in the onset and development of GN by mediating adaptive and innate immunity^2^. CD4^+^ T cell subtypes including effector T-helper 1 (Th1), Th2, Th17 and regulatory T (Treg) cells can be categorized according to their expression of cell surface antigens (e.g. cluster of differentiation (CD) markers), lineage-specific transcription factors (e.g. Forkhead box P3 (Foxp3), T-bet), and cytokines (e.g. interferon (IFN)-γ, IL-4, IL-10, IL-17)^3, 4^. While effector CD4^+^ T cells are pathogenic in immune-related GN, Tregs attenuate renal injury by suppressing the effector T cell-mediated immune response^5^.

Tregs play a crucial role in immune homeostasis and tolerance^6^. Decreased number of, and impaired immunosuppression by, Tregs are associated with many types of kidney diseases, including immune-mediated kidney disease, proteinuric renal disease and acute kidney injury^7^. Whereas deletion of CD4^+^ CD25^+^ Tregs augments renal inflammation, adoptive transfer of CD4^+^ CD25^+^ Tregs attenuates kidney injury in anti-glomerular basement membrane (anti-GBM) GN^8^.

As an immunophenotypic marker more specific to Tregs than CD25 (IL-2 receptor alpha chain), Foxp3 controls the differentiation and immunosuppressive function of Tregs^9–11^. Its pivotal role is demonstrated in the paediatric X-linked multiple organ autoimmune disorder named immunodysregulation polyendocrinopathy enteropathy X-linked syndrome (IPEX), in which mutation in *FOXP3* results in loss of Tregs, severe inflammation, lymphoproliferation, and hyperactive T cell infiltration in multiple organs^12^. This disease phenotype is recapitulated in the X-linked scurfy mouse mutant^13^, and can be rescued by adoptive transfer of Tregs. Furthermore, forced expression of Foxp3 in CD4^+^ CD25^−^ T cells can induce acquisition of the Treg phenotype and immunosuppressive properties *in vitro* ^10^. In models of kidney disease, adoptive transfer of Foxp3-transduced polyclonal T cells protects against chronic renal injury induced by doxorubicin (Adriamycin) *in vivo* ^14^. In contrast, depletion of Foxp3^+^ Tregs induced by diphtheria toxin aggravates T cell-mediated nephrotoxicity and nephritis in Foxp3-diphtheria toxin receptor transgenic mice^15^.

Although CD4^+^ Foxp3^+^ Tregs can confer protection in many kidney diseases, the functional role of transcription factor Foxp3 in GN has not been fully explored, and the mechanism whereby Foxp3 regulates autoimmune kidney disease remains to be elucidated. In this study, we reveal the mechanisms by which Foxp3 suppresses anti-GBM crescentic GN in the Foxp3 transgenic (Tg) mouse.

## Results

### Foxp3 transgenic mice are protected from kidney injury in anti-GBM crescentic GN

**Figure 1:**
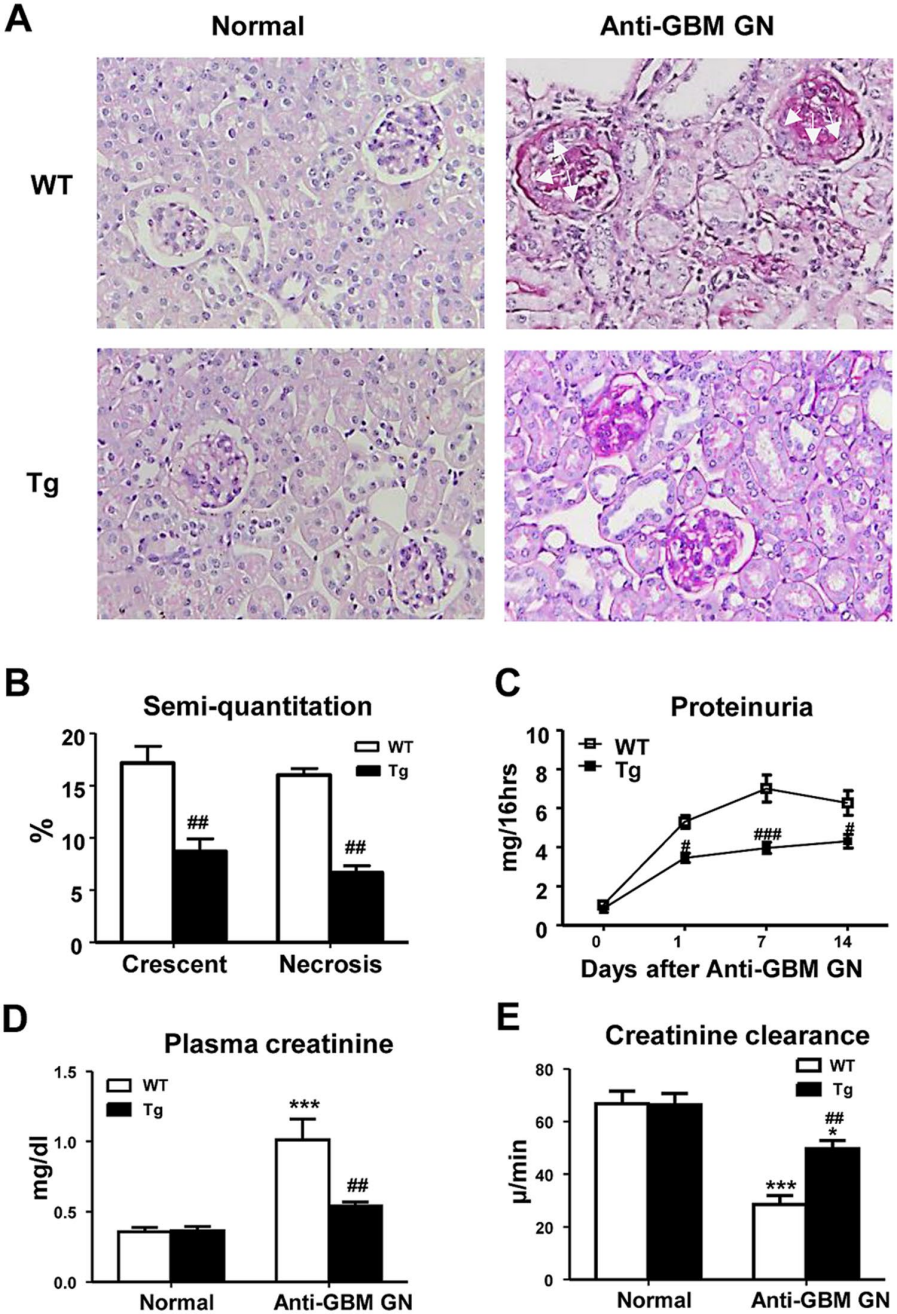
Foxp3 protects from functional and histological kidney injury in anti-GBM GN. (**A**) Representative histology sections stained with PAS. Compared with WT normal mouse kidney, severe glomerular crescent formation, segmental glomerular necrosis and tubulointerstitial damage are evident on Day 14 of anti-GBM disease in WT mice. In contrast, histological damage was substantially ameliorated in Foxp3 Tg mice with anti-GBM disease. Glomerular crescents are indicated by arrows. (**B**) Semi-quantitative analysis of histology. (**C**) Urinary protein excretion. (**D**) Plasma creatinine. (**E**) Creatinine clearance. Each bar represents mean ± SEM. *p < 0.05 and ***p < 0.001 compared with normal mice; ^#^ *p* < 0.05, ^##^ *p* < 0.01 and ^###^ *p* < 0.001 compared with the WT mice. n = 6–8 mice per group. Original magnification X200 (**A**).

No histological abnormalities were detected in the kidneys of control Foxp3 WT and Tg mice. However, in WT mice induced to develop severe crescentic GN, the animals developed marked increase in glomerular crescent formation and necrosis, and severe tubulointerstitial damage with extensive protein cast formation in renal tubules (Fig. 1A). These histological changes were associated with a significant increase in proteinuria and serum creatinine, and a reduction in creatinine clearance (Fig. 1B–E). In contrast, all of these induced histological and functional injuries were significantly attenuated in the Foxp3 Tg mice, resulting in a 50% reduction of glomerular crescents and necrosis, urinary protein excretion, serum creatinine and decline in creatinine clearance (Fig. 1A–E).

### Overexpression of Foxp3 inhibits upregulation of renal IL-1β, TNF-α and MCP-1, and attenuates cell-mediated renal injury in anti-GBM GN

**Figure 2:**
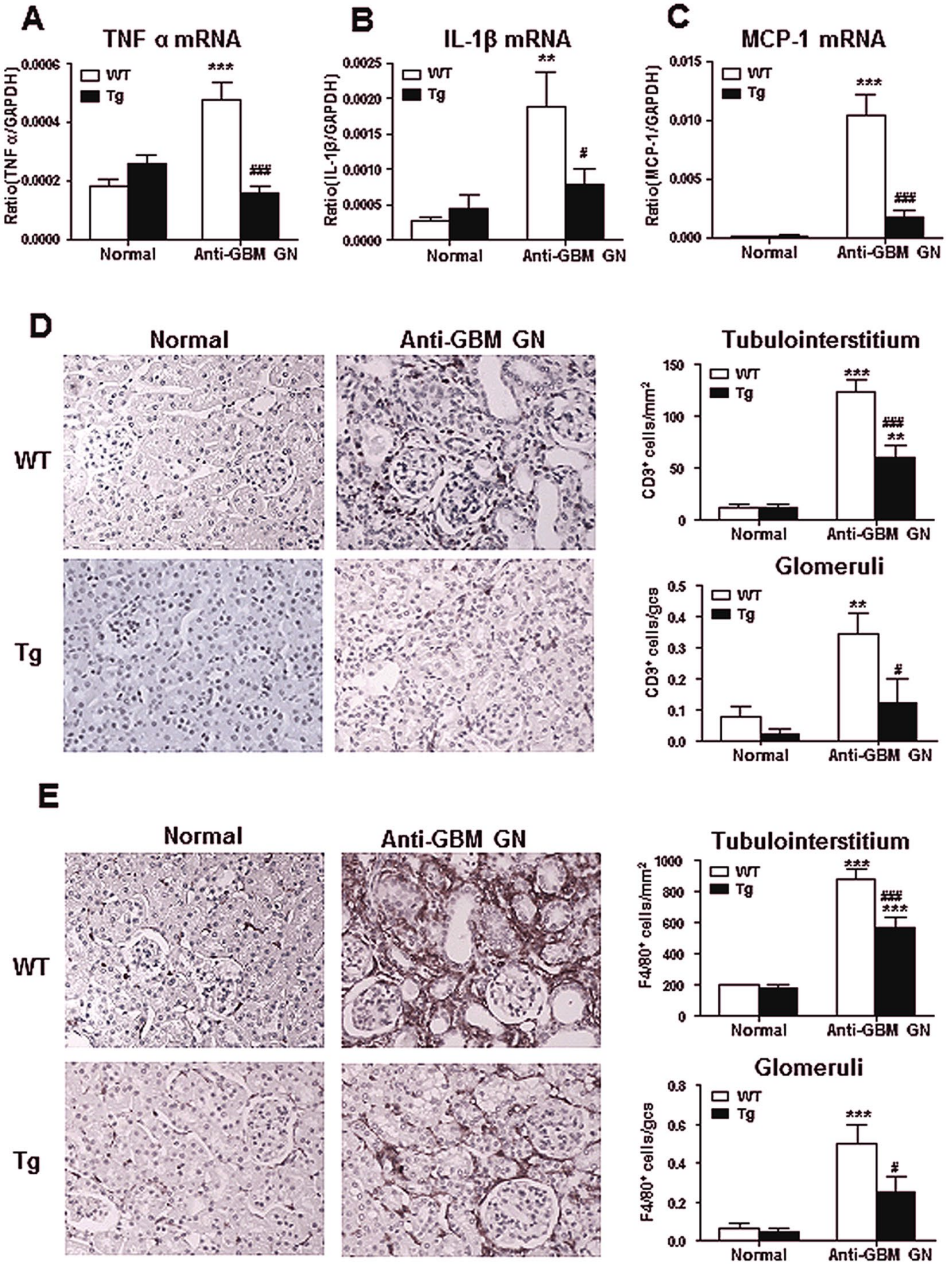
Foxp3 inhibits up-regulation of inflammatory cytokines and cell-mediated kidney injury in anti-GBM GN. Results of Real time PCR show renal inflammatory cytokines ((**A**) TNF-α and (**B)**, IL-1β) and chemokine ((**C**), MCP-1) mRNA expression in normal and anti-GBM GN (day 14) in both WT and Tg mice. (**D**) Immunohistochemistry shows that macrophage (stained for F4/80^+^) infiltration within the kidney with anti-GBM crescentic GN on Day 14 after disease induction is markedly inhibited in Foxp3 Tg mice. (**E**) Immunohistochemistry shows that CD3^+^ T cell (stained with anti-CD3 antibody) infiltration within the kidney with anti-GBM crescentic GN on day 14 is notably inhibited in Foxp3 Tg mice. Each bar represents mean ± SEM. **p < 0.01 and ***p < 0.001 compared with normal mice; ^#^ *p* < 0.05 and ^###^ *p* < 0.001 compared with the WT mice. n = 6–8 mice per group. Original magnification 200X (**D**,**E**).

Real-time PCR demonstrated that WT mice with anti-GBM GN had a marked increase in mRNA levels of IL-1β, TNF-α, and MCP-1 in the diseased kidney (Fig. 2A–C), which was infiltrated by large numbers of CD3^+^ T cells and F4/80^+^ macrophages (Fig. 2D–E). In contrast, mice overexpressing Foxp3 had significantly inhibited upregulation of IL-1β, TNF-α, and MCP-1 mRNAs (Fig. 2A–C) and suppressed CD3^+^ T cell and macrophage infiltration in both glomeruli and tubulointerstitium (Fig. 2D–E).

### Enhancement of Treg but inhibition of Th1, Th2 and Th17 immune responses are mechanisms by which Foxp3 Tg mice protect against anti-GBM crescentic GN

**Figure 3:**
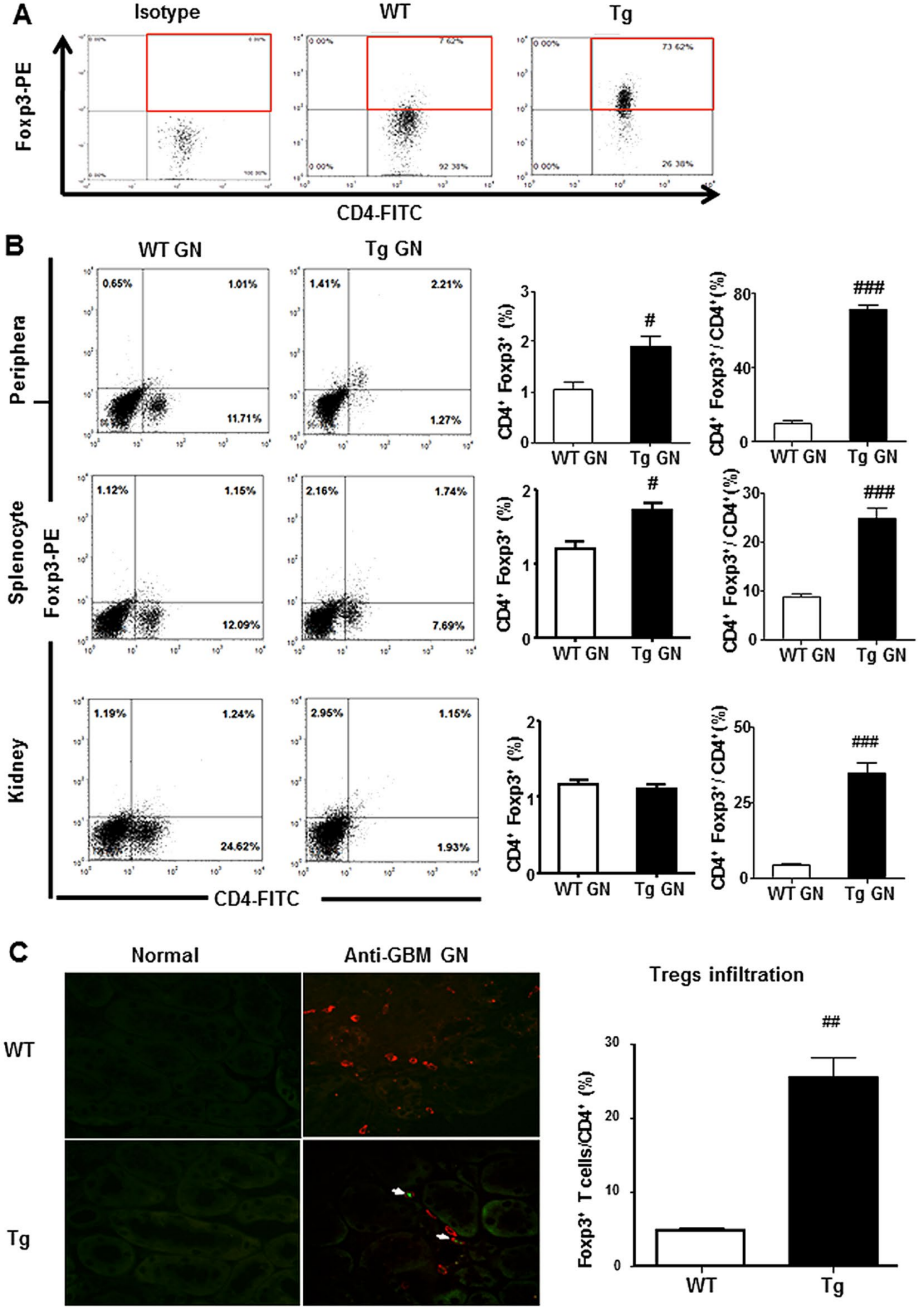
Overexpression of Foxp3 increases proportions of systemic and renal CD4^+^ Foxp3^+^ Tregs. (**A**) Results of two-color flow cytometry show that overexpression of Foxp3 increases proportions of splenic CD4^+^ Foxp3^+^ Tregs in normal Tg mice when compared with WT mice. Gated on CD4^+^ T cells. (**B**) Flow cytometry analysis of peripheral and splenic and renal infiltrated CD4^+^ Foxp3^+^ T cells from diseased WT and Tg mice.(**C**) Infiltration of CD4^+^ Foxp3^+^ Tregs in the inflamed kidney tissues on Day 14 after disease induction was identified by two-color immunofluorescence with Foxp3^+^ (green) and CD4^+^ (red). Examples of CD4^+^ Foxp3^+^ Tregs cells are indicated by white arrows. Each bar represents mean ± SEM. ^##^ *p* < 0.01 compared with the WT mice. n = 3 mice per group. Original magnification 200× (**C**).

**Figure 4:**
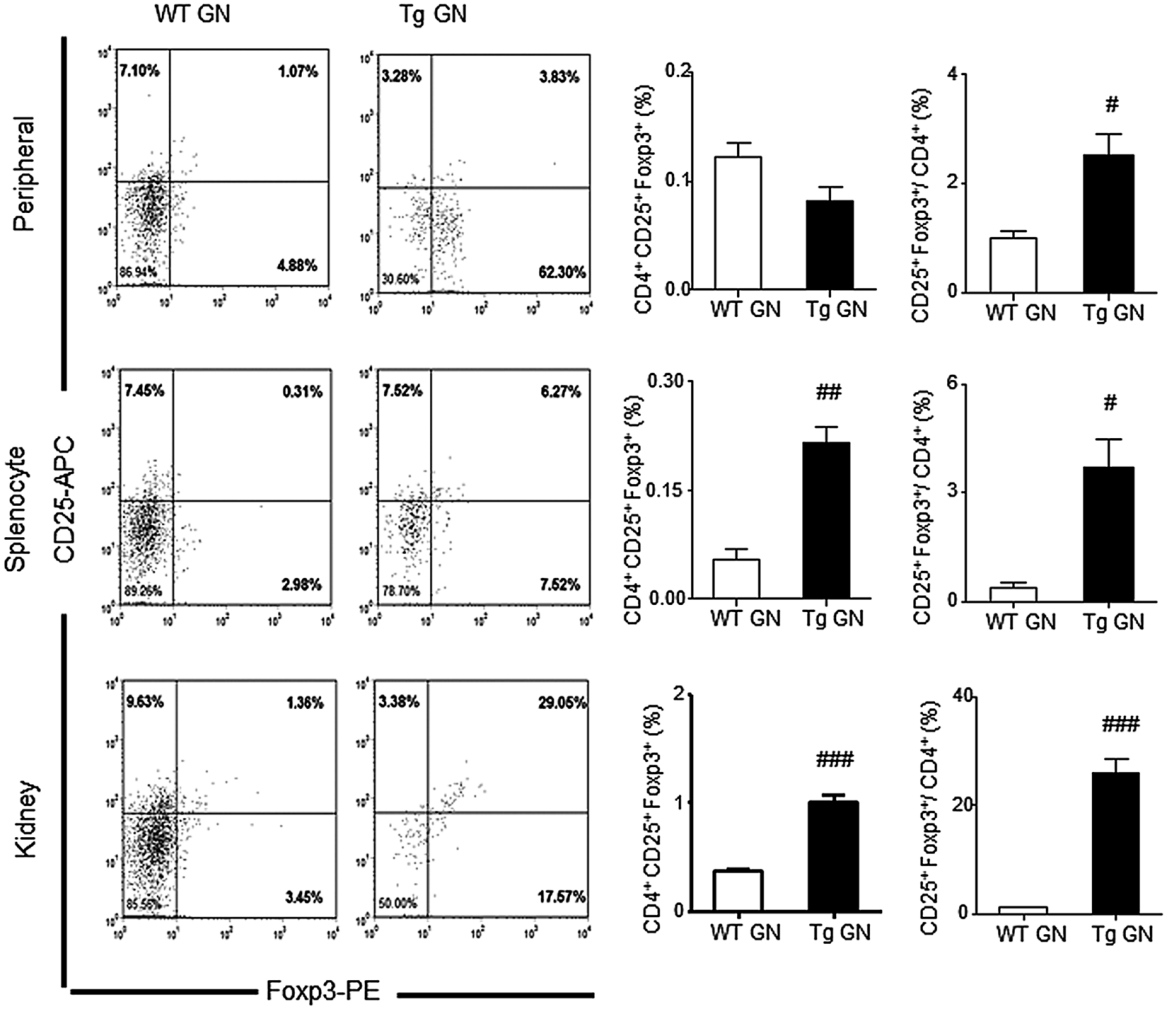
Overexpression of Foxp3 increases CD4^+^ CD25^+^ Foxp3^+^ regulatory T cells in anti-GBM GN. Flow cytometric analysis of peripheral and splenic and renal infiltrated CD4^+^ CD25^+^ Foxp3^+^ Tregs from diseased WT and Tg mice. Gated on CD4^+^ T cells. Each bar represents mean ± SEM. ^#^ *p* < 0.05, ^##^ *p* < 0.01 and ^###^ *p* < 0.001 compared with the WT mice. n = 3 mice per group.

Since Foxp3 is a master transcriptional factor of Treg development^16^, we first profiled the Treg population in both control WT and Foxp3 Tg mice. Using flow cytometry, we demonstrated a 10-fold increase in CD4^+^ Foxp3^+^ immunostaining of cells in the spleen of control Foxp3 Tg mice compared with that of the WT, indicative of an increased ratio of CD4^+^ Foxp3^+^ Tregs to CD4^+^ T cells in the Foxp3 Tg animals (Fig. 3A). Although there were fewer CD4^+^ T cells infiltrating the kidney of Foxp3 Tg mice compared with the WT, 2-colour immunofluorescence histology revealed a 5-fold increase in the frequency of CD4^+^ Foxp3^+^ Tregs in the diseased kidneys of Foxp3 Tg mice (Fig. 3C). Consistent with this finding, the increased frequency but not absolute number of CD4^+^ Foxp3^+^ Tregs locally in the diseased kidney was confirmed by 2-colour flow cytometry (Fig. 3B). Both absolute and relative number of CD4^+^ Foxp3^+^ Tregs was significantly increased systemically in peripheral blood and in the spleen of Foxp3 Tg mice compared with the WT (Fig. 3B). As CD25 was also considered a Treg marker, we analysed the CD4^+^ CD25^+^ Foxp3^+^ population in both diseased WT and Tg mice. Flow cytometric analysis verified a significant increase in the total number and relative ratio of CD4^+^ CD25^+^ Foxp3^+^ Tregs from peripheral blood, the spleen and the diseased kidney in Tg GN mice compared with WT GN mice (Fig. 4).

**Figure 5:**
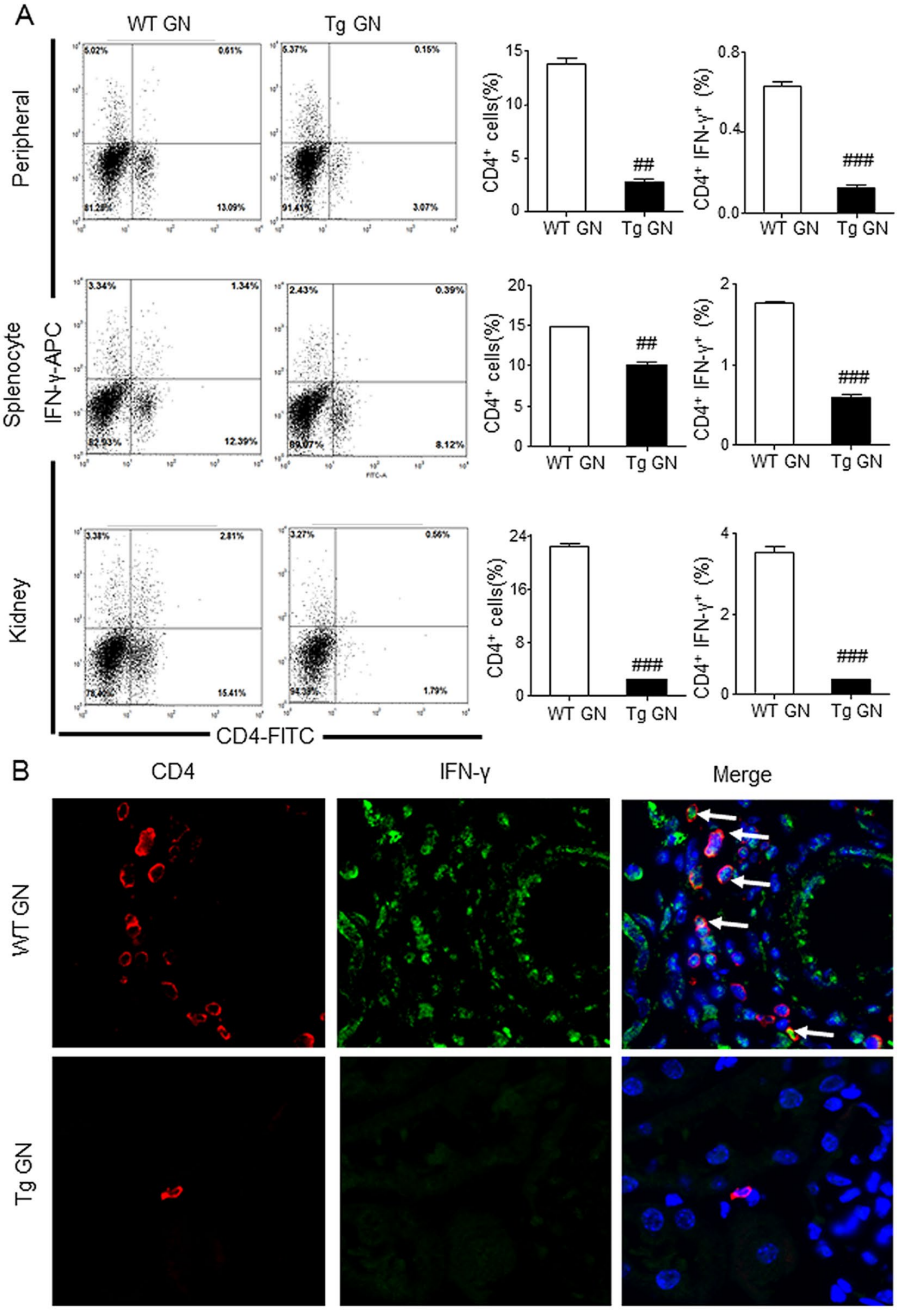
Systemic and renal infiltrated CD4^+^ IFN-γ^+^ Th1 cells are reduced in Foxp3 Tg mice compared with WT mice. (**A**) Flow cytometric analysis of peripheral and splenic and renal infiltrated CD4^+^ IFN-γ^+^ Th1 cells from diseased WT and Tg mice. (**B**) Infiltration of CD4^+^ IFN-γ^+^ Th1 in the inflamed kidney tissues on Day 14 after disease induction was identified by two-color immunofluorescence with IFN-γ^+^ (green) and CD4^+^ (red). Examples of CD4^+^ IFN-γ^+^ Th1 cells are indicated by white arrows. Each bar represents mean ± SEM. ^###^ *p* < 0.001 compared with the WT mice. n = 3 mice per group. Original magnification 400× (**B**).

**Figure 6:**
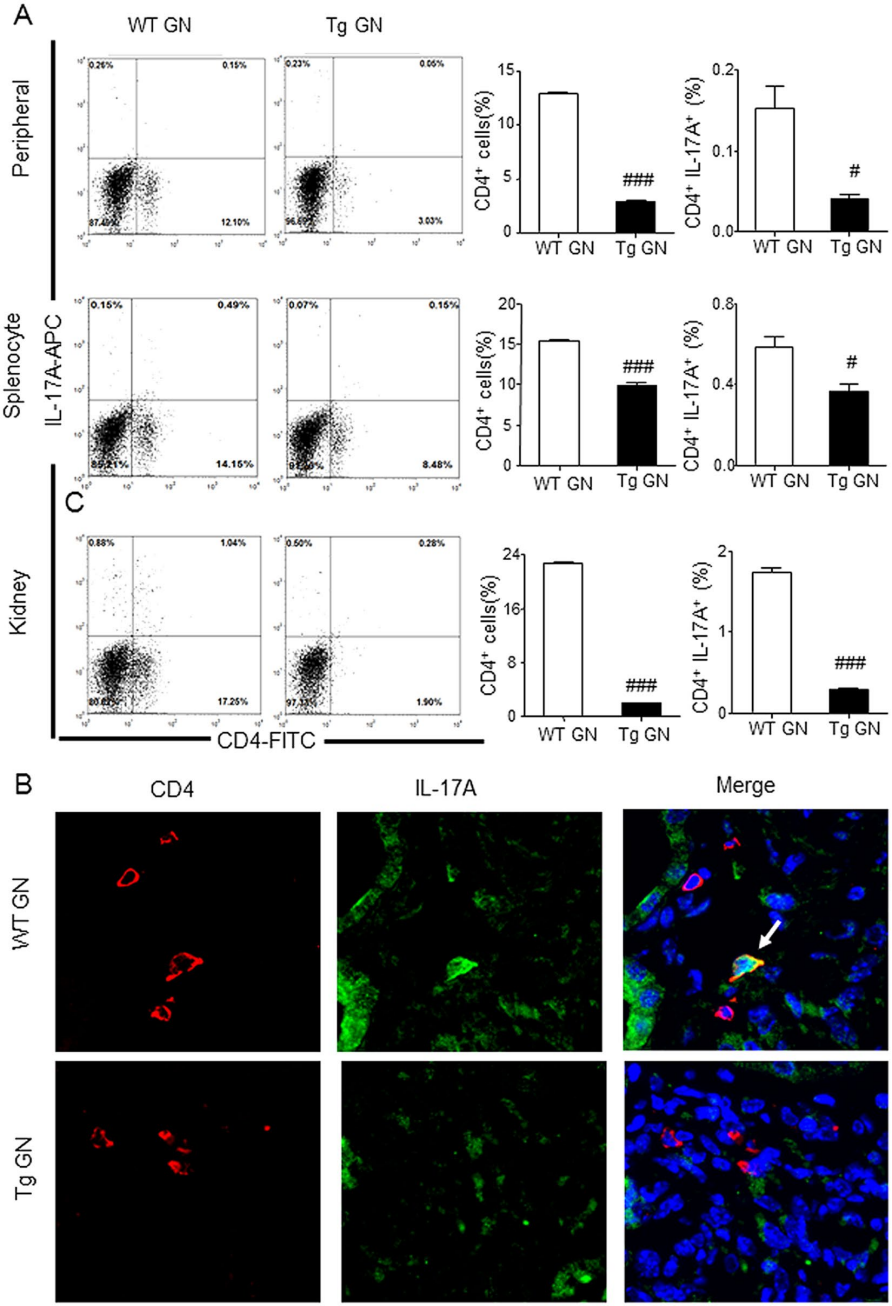
Systemic and renal infiltrated CD4^+^ IL-17A^+^ Th17 cells are decreased in Foxp3 Tg mice when compared with WT mice. (**A**) Flow cytometric analysis of peripheral and splenic and renal infiltrated CD4^+^ IL-17A^+^ Th17 cells from diseased WT and Tg mice. (**B**) Infiltration of CD4^+^ IL-17A^+^ Th17 in the inflamed kidney tissues on Day 14 after disease induction was identified by two-color immunofluorescence with IL-17A^+^ (green) and CD4^+^ (red). Examples of CD4^+^ IL-17A^+^ Th17 cells are indicated by white arrows. Each bar represents mean ± SEM. ^#^ *p* < 0.05 and ^###^ *p* < 0.001 compared with the WT mice. n = 3 mice per group. Original magnification 400X (**B**).

**Figure 7:**
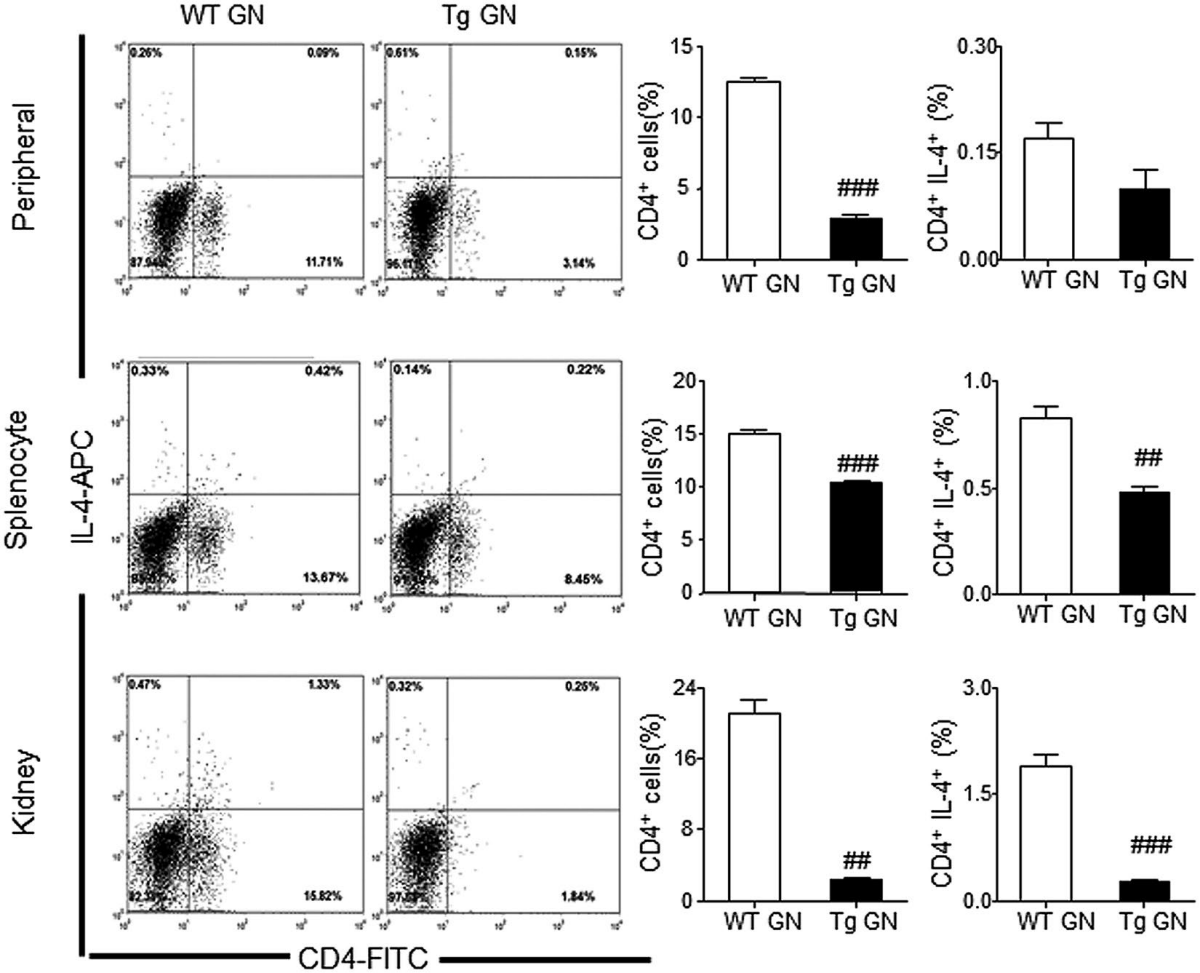
Systemic and renal infiltrated CD4^+^ IL-4^+^ Th2 cells are decreased in Foxp3 Tg mice when compared with WT mice. Flow cytometry analysis of peripheral and splenic and renal infiltrated CD4^+^ IL-4^+^ Th2 cells from diseased WT and Tg mice. Each bar represents mean ± SEM. ^##^ *p* < 0.01 and ^###^ *p* < 0.001 compared with the WT mice. n = 3 mice per group.

Further immunophenotypic analysis suggested that the increase in the number of CD4^+^ Foxp3^+^ Treg cells in Foxp3 Tg mice was associated with fewer CD4^+^ IFN-γ^+^ Th1 cells and fewer CD4^+^ IL-17A^+^ Th17 cells infiltrating the kidney, when compared with the WT mice (Figs 5B and 6B). Flow cytometry enumerated a significant reduction in the number of Th1, Th2 and Th17 cells in Tg GN mice compared with the WT GN mice, both systemically in peripheral blood and the spleen and, locally, in the diseased kidney (Figs 5A, 6A and 7). This significant reduction in the number of Th1, Th2 and Th17 cells in Tg GN mice may result from down-regulation of total CD4^+^ T cells (Figs 5A, 6A and 7).

**Figure 8:**
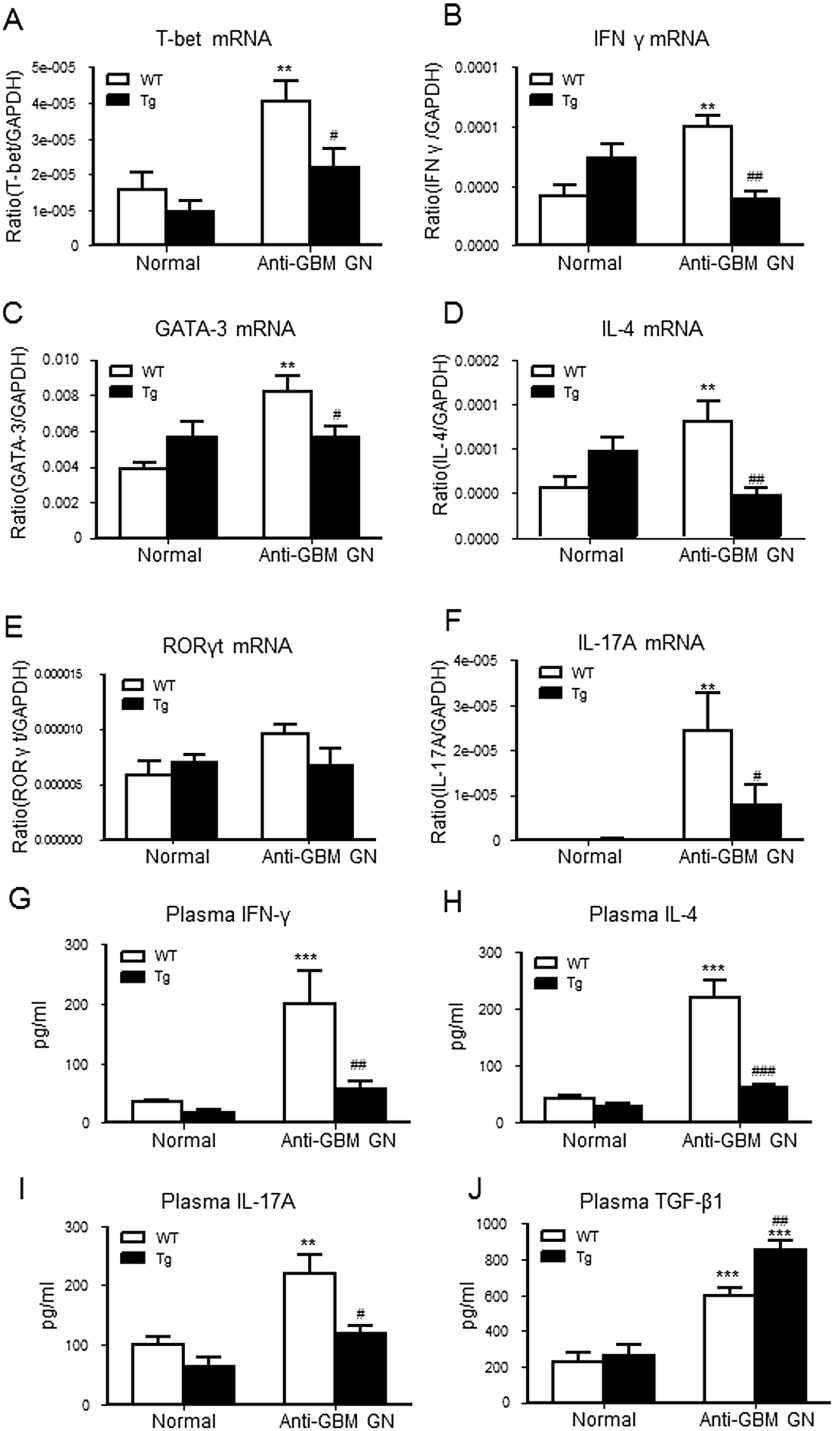
T cell-mediated proinflammatory immune response is attenuated in Foxp3 Tg mice with anti-GBM GN. Total kidney RNA was analysed by real-time PCR for the expression of molecules involved in the Th1 response including: (**A**) T-bet, (**B**) IFN-γ and the Th2 response such as: (**C**) GATA3 and (**D**) IL-4 and the Th17 response such as: (**E**) RORγt and (**F**) IL-17A. Levels of immune cytokines in plasma were measured by ELISA. (**G**) Circulating levels of the Th1 cytokine, IFN-γ. (**H**) Circulating levels of the Th2-cytokine, IL-4. (**I**) Circulating levels of the Th17 cytokine, IL-17A. (**J**) Circulating levels of the immunoregulatory cytokine, TGF-β1. Each bar represents mean ± SEM. **p < 0.01 and ***p < 0.001 compared with normal mice; ^#^ *p* < 0.05 and ^##^ *p* < 0.01 and ^###^ *p* < 0.001 compared with the WT mice. n = 6–8 mice per group.

Real-time PCR also demonstrated that compared with the WT, mice overexpressing Foxp3 had significantly lower mRNA levels of master transcriptional factors for Th1 (T-bet), Th2 (GATA-3), and Th17 (RORγt), and their signature cytokines including IFN-γ, IL-4 and IL-17A, respectively, in the diseased kidney (Fig. 8A–F). These findings were confirmed by ELISA showing significantly higher levels of circulating plasma IFN-γ, IL-4 and IL-17A in the diseased WT mice, contrasting with blunted levels in the Foxp3 Tg mice with GN (Fig. 8G–I). Notably, blood plasma levels of transforming growth factor β1 (TGF-β1) was significantly elevated in the latter animal group (Fig. 8J).

### Over-expression of Foxp3 attenuates plasma anti-sheep IgG antibody production but does not affect the immune complex deposition in inflamed glomeruli

**Figure 9:**
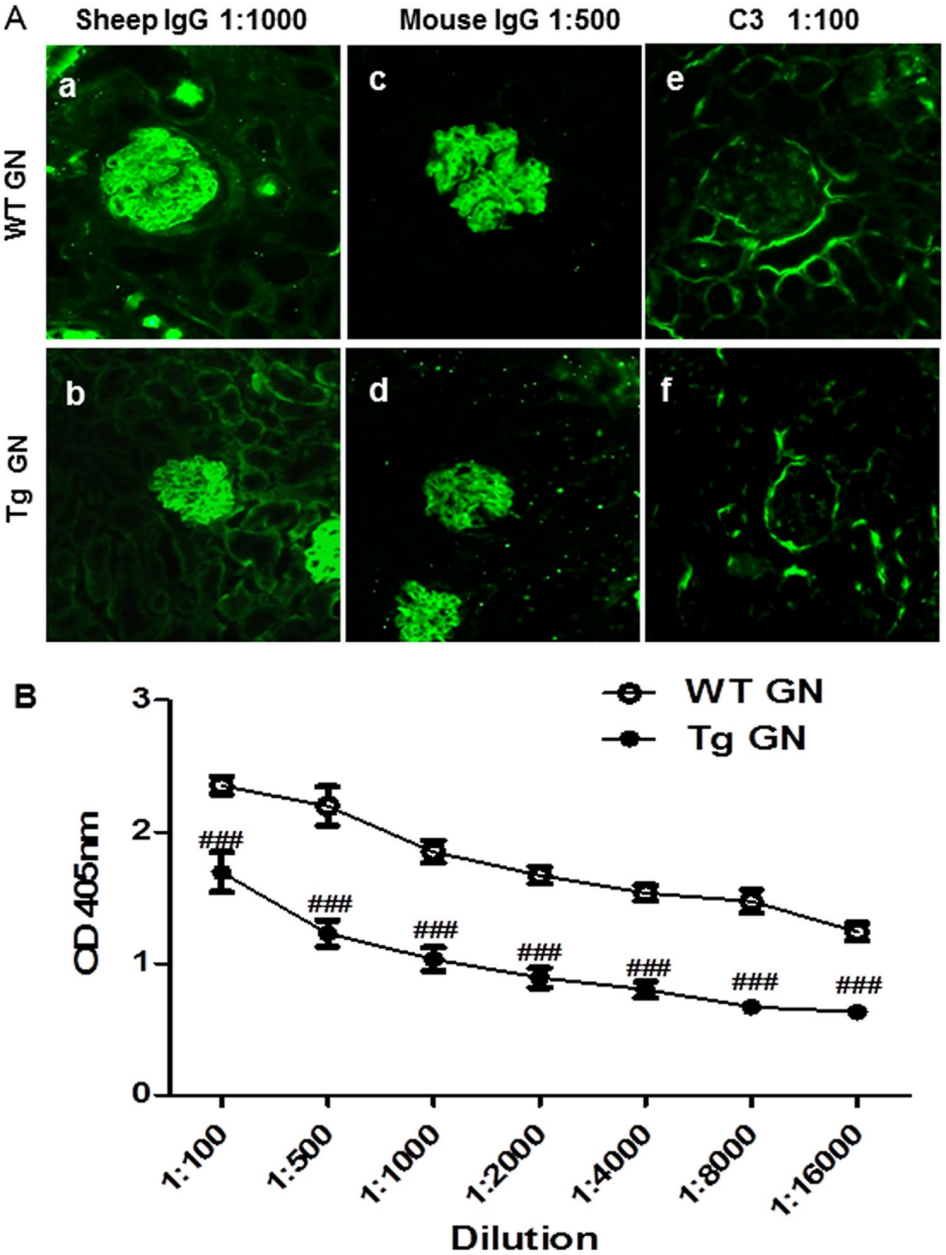
Overexpression of Foxp3 attenuates plasma mouse anti-sheep IgG antibody production but does not affect immune complex deposition in inflamed glomeruli. (**A**) Representative immunofluorescence staining of glomeruli from WT and Tg mice with anti-GBM GN for the deposition of sheep IgG (*a* and *b*), mouse IgG (*c* and *d*) and mouse C3 (*e* and *f*). (**B**) ELISA test of plasma mouse anti-sheep IgG from WT and Tg mice with anti-GBM GN. Each bar represents mean ± SEM. ^###^ *p* < 0.001 compared with the WT mice. n = 6–8 mice per group.

Results of immunofluorescence staining indicated that there was no difference in the glomerular deposition of sheep anti-mouse GBM antibody, mouse IgG, and complement component C3 in the diseased kidney of both WT and Tg mice (Fig. 9A). However, the level of mouse anti-sheep IgG antibodies was significantly attenuated in Tg GN mice compared with WT GN mice (Fig. 9B).

## Discussion

In this study, we report that mice overexpressing Foxp3 are at least partially protected from induced anti-GBM crescentic GN. Foxp3 Tg mice had attenuated (1) glomerular necrosis and crescent formation, (2) proteinuria, (3) plasma creatinine increase, (4) creatinine clearance decline, and (5) renal inflammation mediated by T cells and macrophages and the proinflammatory cytokines (TNF-α, IL-1β and MCP-1). Our further analysis revealed that expansion of the systemic and renal Treg compartment occurred in association with demonstrable diminution of Th1, Th2 and Th17 immune responses, which may be key mechanisms by which Foxp3 exerts protection against kidney injury in anti-GBM crescentic GN.

Foxp3 is essential for mouse Treg development and function, and the immunoregulatory function of Tregs is dependent on Foxp3 expression level^17, 18^. In our study, the Foxp3 Tg mouse strain had approximately 16 copies of the transgene^19^ and had much higher levels of Foxp3 expression confirmed by our findings from flow cytometry, and expression of Foxp3 was restricted to CD4^+^ T cells.

Increased proportion of Tregs alone may not be sufficient to abolish the development of anti-GBM GN; but rather, the disease may be attenuated. It is clear that accelerated anti-GBM disease is initiated by glomerular deposition of immune complex followed by complement activation with accumulation of neutrophils. However, no difference in glomerular deposition of immune complex and complement C3 was found between the WT and the Foxp3 Tg mice with anti-GBM GN. Results from this study indicate that Tregs may not have direct effect on complement-mediated glomerular injury in crescentic GN. Results from other studies including our previous reports demonstrated that T cell-mediated immunity was crucial in anti-GBM disease with or without glomerular deposition of immune complex. Therefore, we postulate that Tregs protect against kidney injury in crescentic GN via targeting effector T cell-mediated immune response as a primary mechanism.

In this study, we observed that increased proportions of systemic and renal Tregs suppressed the Th1 response, evidenced by downregulation of systemic and renal IFN-γ and in correlation with a decrease of glomerular crescent formation, T cell and macrophage infiltration into glomeruli and tubulointerstitium of the renal cortex. But the specific immunosuppressive mechanisms of Tregs preventing GN remains unexplored, as Tregs may also exert immunosuppressive function in cell contact-dependent and regulatory cytokine-dependent manner as reported by other researchers^20^. Our data showed that overexpression of Foxp3 upregulated the plasma level of the immunoregulatory cytokine, TGF-β1. This suggests that Treg cell-derived plasma TGF-β1 (presumably latent TGF-β1) was capable of suppressing immune response without inducing fibrosis locally in the kidney as shown in our previous study in anti-GBM GN induced in TGF-β1 transgenic mice^21^. TGF-β1 potently inhibits T-bet expression in Th1 cells^22^, thereby suppressing levels of IFN-γ in the circulation. Renal downregulation of IFN-γ is also dependent on a fall in T-bet expression. Overexpression of Foxp3 also leads to a downregulation of the Th2 response. In the Foxp3 Tg mouse, augmented TGF-β1 production may chronically downregulate GATA-3 expression in developing Th2 cells^23^, resulting in diminished expression of IL-4 in plasma and kidney. It was thus not surprisingly that Th2-mediated glomerular necrosis was reduced in the Foxp3 Tg mouse. In addition, Tregs appear to be able to concurrently suppress Th1 and Th2 activation by inhibiting IL-2 production via the TGF-β/Smad3 signalling pathway^24^.

Effector Th17 cells contribute to the pathogenesis of proliferative and crescentic GN^25, 26^. Upregulation of the Th17 response promotes proinflammatory cytokine production and enhances kidney injury in GN, supported also by findings from the present study. Downregulation of the Th17 response in the kidneys by Tregs is dependent on suppression of renal Th17 differentiation via inhibiting IL-6 expression^27^. As demonstrated in this study, an alternative Treg-independent, Foxp3-dependent mechanism for suppressing the Th17 response is Foxp3 overexpression, which can directly inhibit Th17 cell differentiation by antagonizing RORγt function^28^.

In summary, we have shown that Foxp3 plays a protective role in crescentic GN by altering the systemic and renal Treg compartment, and importantly, suppresses Th1, Th2, and Th17 immune responses to attenuate inflammation and injury.

## Materials and Methods

### Animal

The Foxp3 Tg mice (strain 2826, C57BL/6 background) were used in this study^13^. Foxp3 Tg genotype was confirmed by PCR analysis in each mouse. Age-matched Foxp3 wildtype (WT) mice also derived from the strain bred in our animal centre. All animals were raised under a specific pathogen-free condition at 25 °C with a normal 12-hour light-and-12-hour dark cycle. Mice were allowed free access to standard food and sterilised water supplied by our animal centre. The experimental procedures were approved by the Animal Experimentation Ethnics Committee at the Chinese University of Hong Kong and all experiments were performed in accordance with relevant guidelines and regulations.

### Mouse model of anti-GBM GN

An accelerated anti-GBM GN model was induced in the littermate male Foxp3 WT and Tg mice (25 g, 8 weeks old) according to a well-established protocol^22, 29^. Briefly, groups of eight Foxp3 WT or Tg mice were firstly immunised by flank subcutaneous injection with normal sheep IgG mixed with Freund’s complete adjuvant (Sigma Aldrich, St. Louis, Missouri, USA) 5 days in advance. Subsequently, these mice were administrated sheep anti-mouse GBM IgG at a dose of 60 μg/g of body weight (termed day 0) via tail vein injection, and were sacrificed by injection of a lethal dose of a ketamine and xylazine mix on day 14. Groups of 6 age-matched normal Foxp3 WT and Tg mice were used as normal control.

### Measurement of proteinuria and creatinine

Urinary protein excretion was collected before and after induction of disease on days 0, 1, 7 and 14, and was examined by using the Coomassie Brilliant Blue method. Plasma and urinary creatinine were tested using an enzymatic kit (Stanbio Laboratory, Boerne, USA) as described previously^22, 30^.

### Histology and immunohistochemistry

Changes in renal morphology were detected in methyl Carnoy’s fixed, paraffin-embedded tissue sections (4 μm) using the periodic acid Schiff (PAS) method. Glomerular crescent formation and necrosis were scored by counting 50 glomeruli on PAS-stained section and expressed as percentage.

Immunohistochemistry was performed in paraffin sections using a microwaved-based antigen retrieval technique^31^. Antibodies used in this study included: rat anti-mouse monoclonal antibody to macrophages (F4/80) (Serotec, Oxford, UK), rabbit polyclonal antibodies to CD3^+^ T cells (SP7) (Abcam, Cambridge, UK). The number of positive cells for CD3 and F4/80 was counted in 20 consecutive glomeruli and expressed as cells per glomerular cross-section (gcs), while positive cells in the tubulointerstitium were counted under high-power fields (400 × magnification) by means of a 0.0625-mm^2^ graticule fitted in the eyepiece of the microscope and expressed as cells per mm^2^.

### Immunofluorescence

Deposition of immune reactants within the glomeruli was determined by direct immunofluorescence with FITC-conjugated polyclonal antibodies to sheep IgG, mouse IgG, and complement C3 as described previously^32^. Tregs infiltrating the kidney were identified in frozen section (4 μm) by 2-color immunofluorescence with FITC-rat anti-mouse Foxp3 monoclonal antibody (eBioscience, San Diego, California, USA) and Dylight 550-rat anti-mouse CD4 monoclonal antibody (Leinco Technologies, St. Louis, Missouri, USA) as described previously^33^. Th1 cells were detected using antibodies against CD4 and IFN-γ (rabbit polyclonal antibody, Abcam, Cambridge, UK), whereas Th17 cells were defined by positive immunostaining against CD4 and IL-17A (rabbit polyclonal antibody, Abcam, Cambridge, UK). Sections were counterstained with DAPI and examined under a Zeiss Axioplan2 imaging microscope (Carl Zeiss, Oberkoche, Germany).

### ELISA

Plasma levels of IFN-γ, IL-4, IL-17A and TGF-β1 were measured by ELISA kits (R&D Systems, Minneapolis, USA) following the manufacturer’s protocol. Quantitation of mouse anti-sheep IgG in plasma was performed as described previously^22, 34^.

### Real-time PCR

Total kidney RNA was isolated using the RNeasy Kit according to the manufacturer’s instructions (Qiagen, Düsseldorf, Germany). cDNA was synthesised and real-time PCR was performed on an Opticon 2 real-time PCR machine (Bio-Rad Laboratories, Hercules, California, USA) using the IQ SYBR Green supermix reagent (Bio-Rad Laboratories) as described previously. The primers used in this study, including mouse TNF-α, IL-1β, MCP-1, T-bet, IFN-γ, RORγt, IL-17A, GATA3, IL-4 and glyceraldehyde-3-phosphate dehydrogenase (GAPDH), were as mentioned previously^33, 34^. The ratio of interested mRNA was normalised to GAPDH mRNA expression.

### Flow cytometry

Single cells were isolated and analysed by flow cytometry as described previously^34, 35^. Briefly, mouse kidney and spleen were digested with blenzyme 4 (0.1 mg/ml, Roche Inc., Indianapolis, Indiana, USA) and DNase I (100 U/ml) into cell suspension. Then lymphocytes were separated by discontinuous density centrifugation on Percoll gradients (40%, 60%, and 80%, Sigma Aldrich, St. Louis, Missouri, USA). After being stimulated with Cell Stimulation Cocktail (plus protein transport inhibitors, eBioscience, San Diego, California, USA), the isolated cells were fixed and permeablised with IC Fixation Buffer and Permeabilization Buffer (eBioscience). Subsequently, these cells were stained with FITC-conjugated anti-mouse CD4, APC-conjugated anti-mouse IFN-γ, IL-4-APC, IL-17-APC, CD25-APC, or PE-conjugated Foxp3. Flow cytometry was performed on a FACS Calibar using the CellQuest Pro Analysis software (BD Biosciences, Franklin Lakes, New Jersey, USA).

### Statistical Analyses

All of the statistical tests were performed using Prism 5.0 GraphPad Software (GraphPad Software, La Jolla, California, USA). Data obtained from this study were expressed as the mean ± SEM. Two-group comparisons were performed using an independent sample t test unless otherwise indicated. Multiple group comparisons were performed using one-way analysis of variance (ANOVA) followed by Tukey’s post hoc tests. Differences with a *p* value less than 0.05 were considered statistically significant.

## References

[1] Chadban SJ, Atkins RC (2005). Glomerulonephritis. Lancet.

[2] Tipping PG, Holdsworth SR (2006). T cells in crescentic glomerulonephritis. J Am Soc Nephrol.

[3] Lazarevic V, Glimcher LH, Lord GM (2013). T-bet: a bridge between innate and adaptive immunity. Nat Rev Immunol.

[4] Campbell DJ, Koch MA (2011). Phenotypical and functional specialization of FOXP3+ regulatory T cells. Nat Rev Immunol.

[5] Hall BM (2015). T Cells: Soldiers and Spies–The Surveillance and Control of Effector T Cells by Regulatory T Cells. Clin J Am Soc Nephrol.

[6] Sakaguchi S, Yamaguchi T, Nomura T, Ono M (2008). Regulatory T cells and immune tolerance. Cell.

[7] Ghali JR, Wang YM, Holdsworth SR, Kitching AR (2016). Regulatory T cells in immune-mediated renal disease. Nephrology (Carlton).

[8] Wolf D (2005). CD4 + CD25+ regulatory T cells inhibit experimental anti-glomerular basement membrane glomerulonephritis in mice. J Am Soc Nephrol.

[9] Hori S, Nomura T, Sakaguchi S (2003). Control of regulatory T cell development by the transcription factor Foxp3. Science.

[10] Khattri R, Cox T, Yasayko SA, Ramsdell F (2003). An essential role for Scurfin in CD4 + CD25+ T regulatory cells. Nat Immunol.

[11] Fontenot JD, Gavin MA, Rudensky AY (2003). Foxp3 programs the development and function of CD4 + CD25+ regulatory T cells. Nat Immunol.

[12] Ramsdell F, Ziegler SF (2014). FOXP3 and scurfy: how it all began. Nat Rev Immunol.

[13] Brunkow ME (2001). Disruption of a new forkhead/winged-helix protein, scurfin, results in the fatal lymphoproliferative disorder of the scurfy mouse. Nat Genet.

[14] Wang YM (2006). Foxp3-transduced polyclonal regulatory T cells protect against chronic renal injury from adriamycin. J Am Soc Nephrol.

[15] Paust HJ (2011). Regulatory T cells control the Th1 immune response in murine crescentic glomerulonephritis. Kidney Int.

[16] Rudensky AY (2011). Regulatory T cells and Foxp3. Immunol Rev.

[17] Chauhan SK, Saban DR, Lee HK, Dana R (2009). Levels of Foxp3 in regulatory T cells reflect their functional status in transplantation. J Immunol.

[18] Wan YY, Flavell RA (2007). Regulatory T-cell functions are subverted and converted owing to attenuated Foxp3 expression. Nature.

[19] Khattri R (2001). The amount of scurfin protein determines peripheral T cell number and responsiveness. J Immunol.

[20] Sakaguchi S, Wing K, Onishi Y, Prieto-Martin P, Yamaguchi T (2009). Regulatory T cells: how do they suppress immune responses?. Int Immunol.

[21] Huang XR, Chung AC, Zhou L, Wang XJ, Lan HY (2008). Latent TGF-beta1 protects against crescentic glomerulonephritis. J Am Soc Nephrol.

[22] Gorelik L, Constant S, Flavell RA (2002). Mechanism of transforming growth factor beta-induced inhibition of T helper type 1 differentiation. J Exp Med.

[23] Gorelik L, Fields PE, Flavell RA (2000). Cutting edge: TGF-beta inhibits Th type 2 development through inhibition of GATA-3 expression. J Immunol.

[24] McKarns SC, Schwartz RH, Kaminski NE (2004). Smad3 is essential for TGF-beta 1 to suppress IL-2 production and TCR-induced proliferation, but not IL-2-induced proliferation. J Immunol.

[25] Summers SA (2009). Th1 and Th17 cells induce proliferative glomerulonephritis. J Am Soc Nephrol.

[26] Paust HJ (2012). Chemokines play a critical role in the cross-regulation of Th1 and Th17 immune responses in murine crescentic glomerulonephritis. Kidney Int.

[27] McGovern JL (2012). Th17 cells are restrained by Treg cells via the inhibition of interleukin-6 in patients with rheumatoid arthritis responding to anti-tumor necrosis factor antibody therapy. Arthritis Rheum.

[28] Zhou L (2008). TGF-beta-induced Foxp3 inhibits T(H)17 cell differentiation by antagonizing RORgammat function. Nature.

[29] Lv J (2013). Ribosomal protein S19 is a novel therapeutic agent in inflammatory kidney disease. Clin Sci (Lond).

[30] Hou CC (2005). Ultrasound-microbubble-mediated gene transfer of inducible Smad7 blocks transforming growth factor-beta signaling and fibrosis in rat remnant kidney. Am J Pathol.

[31] Lan HY, Mu W, Nikolic-Paterson DJ, Atkins RC (1995). A novel, simple, reliable, and sensitive method for multiple immunoenzyme staining: use of microwave oven heating to block antibody crossreactivity and retrieve antigens. J Histochem Cytochem.

[32] Lan HY (1997). The pathogenic role of macrophage migration inhibitory factor in immunologically induced kidney disease in the rat. J Exp Med.

[33] Tang YJ (2014). Latent transforming growth factor-beta1 protects against bleomycin-induced lung injury in mice. Am J Respir Cell Mol Biol.

[34] Wang YY (2015). Deletion of Smad3 improves cardiac allograft rejection in mice. Oncotarget.

[35] Meng XM (2016). Inflammatory macrophages can transdifferentiate into myofibroblasts during renal fibrosis. Cell Death Dis.

